# Stable Isotope Enrichment (Δ^15^N) in the Predatory Flower Bug (*Orius majusculus*) Predicts Fitness-Related Differences between Diets

**DOI:** 10.3390/insects11040255

**Published:** 2020-04-20

**Authors:** Marta Montoro, Per M. Jensen, Lene Sigsgaard

**Affiliations:** Department of Plant and Environmental Sciences, Section of Organismal Biology, University of Copenhagen, 1871 Frederiksberg C, Denmark; pmj@plen.ku.dk (P.M.J.); les@plen.ku.dk (L.S.)

**Keywords:** nitrogen, carbon, isotope, isotope enrichment, isotope discrimination, diet quality, Heteroptera, rearing

## Abstract

Mass rearing of insects, used both as biological control agents and for food and feed, is receiving increasing attention. Efforts are being made to improve diets that are currently in use, and to identify alternative diets, as is the case with the predatory flower bug (*Orius majusculus*) and other heteropteran predators, due to the high costs of their current diet, the eggs of the Mediterranean flour moth (*E. kuehniella*). The assessment of alternative diets may include measurements of the predator’s fitness-related traits (development time, weight, etc.), and biochemical analyses such as lipid and protein content in the diet and the insects. However, assessing diet quality via the predator’s fitness-related traits is laborious, and biochemical composition is often difficult to relate to the measured traits. Isotope analysis, previously used for diet reconstruction studies, can also serve as a tool for the assessment of diet quality. Here, the variation in discrimination factors or isotope enrichment (Δ^15^N and Δ^13^C) indicates the difference in isotopic ratio between the insect and its diet. In this study, we investigated the link between Δ^15^N and diet quality in the predatory bug *Orius majusculus*. Three groups of bugs were fed different diets: *Ephestia kuehniella* eggs, protein-rich *Drosophila melanogaster* and lipid-rich *D. melanogaster*. The isotopic enrichment and fitness-related measurements were assessed for each group. Results show a relation between Δ^15^N and fitness-related measurements, which conform to the idea that lower Δ^15^N indicates a higher diet quality.

## 1. Introduction

There is a growing body of studies looking into alternative diets for the mass-rearing of predators used in biological control [1,2,3,4,5,6,7]. The fitness-related measurements used as reference for diet quality (development time, weight, etc.) are often complemented by biochemical analyses of the body of the predator and/or the prey [1,2,3,4,5,6,7,8,9]. The information on basic components (nitrogen, lipids and carbohydrates) of a given diet can indicate how nutritionally balanced the diet is [10]. Likewise, the nutritional composition of the entomophagous insects can give an indication of the lack or excess of a particular nutrient [3,4,11,12,13]. However, such analyses do not always show clear patterns which relate to diet quality [2,5,6,7,14]. Often, further complex analyses, that assess minerals, fatty acids, etc., are required [1,2,3,4,8].

Stable Isotope Analysis has been widely used in trophic studies for diet reconstruction, and also has potential as a tool for assessing diet quality [15,16,17,18,19]. In diet quality assessments, the quality is inferred from isotope enrichment (also referred to as isotope discrimination or fractionation). Isotope enrichment (Δ^15^N and Δ^13^C) refers to the difference in carbon and nitrogen isotopic ratios (δ^15^N and δ^13^C) between an animal and its diet [19]. For carbon, the enrichment between the diet and consumer is assumed to be low (~1‰); the carbon isotopic ratio is therefore indicative of the source of carbon in the diet (C3, C4 or marine plants) [15,20]. In contrast, δ^15^N is known to increase with the trophic level by ~3–4‰, and thus predators are typically enriched relative to their diets [15,20]. The enrichment is caused by the discrimination of heavy isotopes, derived from a combination of biochemical and physiological processes, leading to a faster excretion of the lighter isotopes [17,21]. In relation to diet quality, Webb et al. (1998) reported higher Δ^15^N values linked to the consumption of lower quality diets in locusts (*Locusta migratoria*) [22], which conforms to the idea that a high-quality diet is metabolized with fewer physiological processes than a low-quality diet. However, varying allocation patterns between age and sex may affect enrichment patterns [23]. Thus, Oelbermann and Scheu (2002) attribute increased enrichment as associated with increased diet quality in the spider *Pardosa lugubris* (Walckenaer) (Aranae: Lycosidae) to the differences in age and size of the spiderlings [24]. 

The aim of this study was to investigate whether isotope enrichment, and Δ^15^N in particular, is a good indicator of diet quality in the predatory bug *Orius majusculus* (Reuter) (Heteroptera: Anthocoridae), which is currently mass-reared for its use in biological control. For that purpose, we performed stable isotope analysis on one-day-old adults that had been reared on three different diets: *Ephestia kuehniella* (Zeller) (Lepidoptera: Pyralidae) eggs, protein-rich *Drosophila melanogaster* (Meigen) (Diptera: Drosophilidae) and lipid-rich *D. melanogaster*. The three diets were of different quality according to fitness-related traits measured in Montoro et al. (2020a) [6]. We expected to find a negative relationship between Δ^15^N and diet quality. We furthermore provide the results regarding Δ^13^C. In principle, we would expect Δ^13^C to follow the trends of Δ^15^N. However, a diet-induced change in energy metabolism substrates (protein, carbohydrates and fat), and/or related differences in fat and carbohydrate deposition, could lead to differences in Δ^13^C, which would then differ from those observed for Δ^15^N.

## 2. Materials and Methods

### 2.1. Experimental Design

Predator and prey organisms used for the stable isotope analysis were re-sampled from Montoro et al. (2020a) [6]. In the study, first instars of the predator *O. majusculus* (<24 h old) were reared throughout their development on one of three different diets (prey organisms): *Ephestia kuehniella* eggs, protein-rich *D. melanogaster* and lipid-rich *D. melanogaster*, all provided in surplus. Frozen *E. kuehniella* eggs were supplied by EWH BioProduction (Tappernøje, Denmark). *Drosophila melanogaster* were reared on artificial diets enriched with either proteins or lipids as described in Jensen et al. 2010 to produce flies of different composition [25]. Protein-rich *D. melanogaster* cultures were reared with a medium containing a ratio of 3:2 casein (Sigma C-5890, Sigma-Aldrich, Steinheim, Germany) and basic medium (Carolina Instant Drosophila Medium Formula 4-24, Burlington, NC, USA). To produce lipid-rich *D. melanogaster*, the medium contained a ratio of 1:4 sucrose (Sigma 84097, Sigma-Aldrich, Steinheim, Germany) and basic medium (Carolina Instant Drosophila Medium Formula 4-24, Burlington, NC, USA). All *D. melanogaster* cultures were reared at the University of Copenhagen laboratory in plastic bottles of 6 cm diameter at 21 ± 0.5 °C with a photoperiod of 12:12 (L:D). Each plastic bottle contained 15 g of the designated medium mixed with water and 13 grains of yeast.

The diets offered were of different quality for the predator according to several parameters analyzed in Montoro et al. (2020a) [6]. In the current study, we present as quality parameters the proportion of females laying eggs and the developmental speed of the predators. Developmental speed was calculated as: 1/Total development time. We randomly selected 10 male and 10 female *O. majusculus* per treatment for isotope analysis. They were freeze killed at −20 °C, dried at 50 °C for 48 h and then weighed on a Mettler Toledo XP6 balance (Mettler Toledo, Glostrup, Denmark) one day after becoming adults. Due to minimum weight restrictions for the isotope analysis, two *O. majusculus* were pooled per sample leading to a total of five samples per sex and treatment (n = 30). For the diet analysis, six samples of *E. kuehniella* eggs, seven of protein-rich *D. melanogaster* and eight of lipid-rich *D. melanogaster* were selected (n = 21). *Drosophila melanogaster* samples consisted of a mix of 50:50 males and females, mirroring the mix given to the predators as diet [6]. *Ephestia kuehniella* egg samples were a clutch of eggs weighing approximately 1.16 ± 0.04 mg per sample. (Supplementary Tables S1 and S2).

### 2.2. Stable Isotope Analysis

The 15N/14N and 13C/12C in the samples was analyzed at the University of California-Davis Stable Isotope facility on a PDZ Europa ANCA GSL elemental analyzer (Elementar Analysensysteme GmbH, Hanau, Germany) interfaced with a PDZ Europa 20-20 isotope ratio mass spectrometer (IRMS) (Sercon Ltd., Cheshire, United Kingdom). Briefly, the samples are combusted in the elemental analyzer and the produced gases (first N_2_, then CO_2_) are sent to the IRMS in a helium carrier. During analysis, samples are interspersed with different replicates of reference materials. Values are reported as δ^15^N and δ^13^C, and expressed in parts per thousand (‰) according to the relationship:

δ = [(R_sample_ − R_standard_)/R_standard_] × 10^3^, (1)

where R is the ratio of heavy-to-light isotopes in the sample (R_sample_) and in the relevant international standard (R_standard_). The international standard for carbon is VPDB (Vienna Pee Dee Belemnite) and Air for nitrogen. The standard deviation of the measurements is 0.2‰ for δ^13^C and 0.3‰ for δ^15^N [26]. Δ^15^N and Δ^13^C were calculated as the difference in δ^15^N and δ^13^C between the diet and the predator [19,24].

### 2.3. Statistical Analysis

All statistical analyses were performed in R v3.2.5 [27]. Model residuals were explored graphically to adhere to the criteria of normality and homoscedasticity for linear model analysis. The effect of diet and sex on δ^15^N, δ^13^C, Δ^15^N and Δ^13^C was analyzed within the linear models. P-values are provided for multiple comparison analyses with Bonferroni adjustment.

## 3. Results and Discussion

There were significant differences in δ^15^N and δ^13^C between *O. majusculus* fed the three different diets (δ^15^N: *F*_(2,26)_ = 1804.05, *p* < 0.001; δ13C: *F*_(2,26)_ = 1820.96, *p* < 0.001) (Table 1). In the case of δ^15^N, sex also had an effect (*F*_(1,26)_ = 17.31, *p* < 0.001), and males had higher δ^15^N level than females across treatments (t-value = 4.16, *p* < 0.001, n = 30).

Diet also had an impact on isotope enrichment for both Δ^15^N and Δ^13^C (Δ^15^N: F_(2,26)_ = 60.00, *p* < 0.001; Δ^13^C: F_(2,26)_ = 34.69, *p* < 0.001). The Δ^15^N value was higher in the predators fed a lipid-rich *D. melanogaster* diet, followed by those fed the protein-rich *D. melanogaster* and *E. kuehniella* eggs (Table 1). Furthermore, Δ^15^N was higher in males than in females (t-value = 4.16, *p* < 0.001, n = 30). The Δ^13^C variations among diets followed a different pattern than Δ^15^N. The lowest Δ^13^C was found in the predators fed the protein-rich *D. melanogaster*, and there were no significant differences between predators fed *E. kuehniella* eggs and lipid-rich *D. melanogaster* (Table 1). Furthermore, there was no difference in Δ^13^C between sexes.

**Figure 1:**
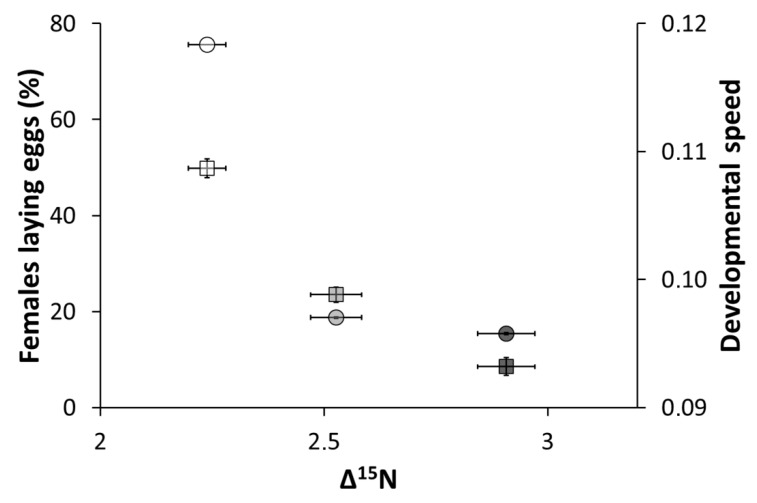
Proportion of females laying eggs (±SE) and developmental speed (±SE) of *O. majusculus* according to the nitrogen isotope enrichment (Δ^15^N ± SE). Each Δ^15^N value corresponds to a different diet: in white, *E. kuehniella* eggs; in grey, protein-rich *D. melanogaster*; and in black, lipid-rich *D. melanogaster*. The circles show the percentage of females laying eggs and the squares the developmental speed. Data on females laying eggs and developmental speed come from Montoro et al. (2020) [6].

This study shows that stable isotope values (δ^15^N, δ^13^C) and isotope enrichment (Δ^15^N, Δ^13^C) change significantly with diet. The values of isotope enrichment corresponded to those observed in previous studies on invertebrates, i.e., ~1‰ for Δ^13^C and ~3–4‰ for Δ^15^N [15,20,22,24,28]. Importantly, the differences in δ^15^N between the diets (prey organisms) and the predator (*O. majusculus*) related directly to group differences in fitness parameters, such as those given by the proportion of females laying eggs and developmental speed (See Montoro et al. 2020a [6]) (Figure 1). Lower quality diets (characterized by lower developmental speed and a lower number of females laying eggs) were associated with higher Δ^15^N, compared to higher quality diets. These findings are in line with the results from Webb et al. (1998) and Adams and Sterner (2000) [22,29], confirming that a high-quality diet is metabolized with fewer physiological processes than a low-quality diet. The nitrogen enrichment was thus most pronounced for the *O. majusculus* reared on lipid-rich *D. melanogaster*, followed by those fed on protein-rich *D. melanogaster* and finally those fed on *E. kuehniella* eggs (the highest quality diet). 

Webb et al. (1998) and Oelbermann and Scheu (2002) reported increased Δ^13^C values in locusts (*L. migratoria*) and spiderlings of *P. lugubris*, respectively, when fed low-quality food [22,24], however they found no clear overall trend relating to prey quality. Similarly, in our study Δ^13^C also varied with diet but did not relate to food quality as Δ^15^N did. The highest Δ^13^C values were recorded for predators fed on *E. kuehniella* eggs and on lipid-rich *D. melanogaster*, representing the highest and lowest quality diets, respectively. We therefore note that Δ^13^C results may also in this case involve ambiguities, as have been pointed out in a previous study [30]. The contrast between Δ^15^N and Δ^13^C, however, would also allow the speculation that differences in the quality of the three diets could result from differences in amino acid composition and protein quality [31]. 

## 4. Conclusions

Our results demonstrate that conducting an analysis of the ^15^N stable isotope can be particularly useful for studies investigating diet quality. In the case of *O. majusculus*, the Δ^15^N values change significantly with diet quality, as higher Δ^15^N values were recorded in lower quality diets when compared to higher quality diets. These changes relate to the fitness-traits measured in Montoro et al. (2020a) more clearly than the measurements of total carbon, nitrogen and lipid, and the carbon-to-nitrogen ratio, did [6]. The simplicity and the low sample size required for the method suggests that this would be an uncomplicated alternative to assess diet quality. The Δ^13^C values also varied significantly with diet, but the changes did not relate to food quality as the Δ^15^N did.

## Figures and Tables

**Table 1:** δ^15^N, δ^13^C, Δ^15^N and Δ^13^C values (mean ± SE) of *O. majusculus* reared on three different diets: *E. kuehniella* eggs (*E. kuehniella*), protein-rich *D. melanogaster* (P-rich flies) and lipid-rich *D. melanogaster* (L-rich flies).

Diet	Parameters			
	δ^15^N (‰)	δ^13^C (‰)	Δ^15^N (‰)	Δ^13^C (‰)
*E. kuehniella*	6.40 ± 0.04a	−28.38 ± 0.06a	2.24 ± 0.04c	0.64 ± 0.06a
P-rich flies	6.06 ± 0.06a	−23.46 ± 0.04b	2.53 ± 0.06b	−0.02 ± 0.06b
L-rich flies	2.52 ± 0.06b	−23.91 ± 0.06b	2.91 ± 0.06a	0.44 ± 0.04a

a–c: Different letters within a column indicate significant between-group differences (*p* < 0.001).

## Supplementary Materials

The research data is found in supplementary materials. The following are available online at https://www.mdpi.com/2075-4450/11/4/255/s1, Table S1: Isotope Data; Table S2: Enrichment Data.
