# Supplementary material for: Stable Isotope Enrichment (Δ15N) in the Predatory Flower Bug (Orius majusculus) Predicts Fitness-Related Differences between Diets
Source: Insects. 2020 Apr 20;11(4):255. doi: 10.3390/insects11040255 (PMC7240723; doi:10.3390/insects11040255)
Supplement: Supplementary file 1 [file insects-11-00255-s001.pdf]

Communication

Stable Isotope Enrichment ($\Delta^{15}N$) in the Predatory Flower Bug (*Orius majusculus*) Predicts Fitness-Related Differences between Diets

Marta Montoro *, Per M. Jensen and Lene Sigsgaard

Department of Plant and Environmental Sciences, Section of Organismal Biology, University of Copenhagen, Frederiksberg C 1871, Denmark; pmj@plen.ku.dk, (P.M.J); les@plen.ku.dk (L.S.)
* Correspondence: mrt.montoro@gmail.com

Table S1. Isotope Data.

Treatment	ID	Sex	$\delta^{13}C$	$\delta^{15}N$	Amount Sample (mg)
Ephestia-Diet	1	NA	–28.35	6.56	1.194
Ephestia-Diet	2	NA	–28.49	6.27	1.168
Ephestia-Diet	3	NA	–28.39	6.34	1.106
Ephestia-Diet	4	NA	–28.34	6.30	1.322
Ephestia-Diet	5	NA	–28.37	6.47	1.078
Ephestia-Diet	6	NA	–28.37	6.46	1.089
Lipid-Diet	1	NA	–23.95	2.35	0.768
Lipid-Diet	2	NA	–24.26	2.56	0.763
Lipid-Diet	3	NA	–23.55	2.64	0.684
Lipid-Diet	4	NA	–24.31	2.57	0.752
Lipid-Diet	5	NA	–24.30	2.44	0.804
Lipid-Diet	6	NA	–23.67	2.26	0.781
Lipid-Diet	7	NA	–22.91	2.83	0.662
Lipid-Diet	8	NA	–24.31	2.52	0.723
Protein-Diet	1	NA	–23.26	6.36	0.983
Protein-Diet	2	NA	–23.40	6.13	0.919
Protein-Diet	3	NA	–23.71	5.67	1.125
Protein-Diet	4	NA	–23.66	5.44	0.908
Protein-Diet	5	NA	–23.58	6.21	0.943
Protein-Diet	6	NA	–23.37	6.36	0.929
Protein-Diet	7	NA	–23.22	6.27	0.763
Ephestia-Predator	1	M	–27.58	8.79	0.302
Ephestia-Predator	2	M	–27.42	8.89	0.338
Ephestia-Predator	3	M	–27.55	8.47	0.31
Ephestia-Predator	4	M	–27.64	8.72	0.34
Ephestia-Predator	5	M	–27.71	8.69	0.36
Ephestia-Predator	1	F	–27.94	8.54	0.54
Ephestia-Predator	2	F	–27.90	8.51	0.53
Ephestia-Predator	3	F	–27.90	8.61	0.53
Ephestia-Predator	4	F	–27.80	8.64	0.51
Ephestia-Predator	5	F	–28.02	8.54	0.52
Lipid-Predator	1	M	–23.73	5.60	0.24
Lipid-Predator	2	M	–23.45	5.31	0.26
Lipid-Predator	3	M	–23.46	5.78	0.26
Lipid-Predator	4	M	–23.73	5.43	0.27
Lipid-Predator	5	M	–23.39	5.65	0.276
Lipid-Predator	1	F	–23.68	5.31	0.48
Lipid-Predator	2	F	–23.19	5.20	0.443
Lipid-Predator	3	F	–23.31	5.30	0.36

Lipid-Predator	4	F	–23.55	5.20	0.485
Lipid-Predator	5	F	–23.20	5.51	0.471
Protein-Predator	1	M	–23.65	8.78	0.31
Protein-Predator	2	M	–23.55	8.61	0.31
Protein-Predator	3	M	–23.30	8.86	0.27
Protein-Predator	4	M	–23.49	8.71	0.28
Protein-Predator	5	M	–23.41	8.55	0.24
Protein-Predator	1	F	–23.68	8.67	0.45
Protein-Predator	2	F	–23.46	8.61	0.42
Protein-Predator	3	F	–23.24	8.44	0.42
Protein-Predator	4	F	–23.47	8.32	0.44
Protein-Predator	5	F	–23.57	8.34	0.43

Table S2. Enrichment Data.

Treatment	ID	Sex	$\Delta^{15}N$	$\Delta^{13}C$
Ephestia	1	F	2.14	0.44
Ephestia	2	F	2.11	0.48
Ephestia	3	F	2.21	0.48
Ephestia	4	F	2.24	0.58
Ephestia	5	F	2.13	0.37
Ephestia	1	M	2.39	0.80
Ephestia	2	M	2.49	0.96
Ephestia	3	M	2.07	0.84
Ephestia	4	M	2.32	0.75
Ephestia	5	M	2.28	0.67
Lipid	1	F	2.79	0.23
Lipid	2	F	2.68	0.72
Lipid	3	F	2.78	0.60
Lipid	4	F	2.68	0.36
Lipid	5	F	2.99	0.71
Lipid	1	M	3.08	0.18
Lipid	2	M	2.79	0.46
Lipid	3	M	3.26	0.45
Lipid	4	M	2.91	0.18
Lipid	5	M	3.13	0.52
Protein	1	F	2.60	–0.22
Protein	2	F	2.55	0.00
Protein	3	F	2.38	0.21
Protein	4	F	2.26	–0.02
Protein	5	F	2.28	–0.11
Protein	1	M	2.72	–0.19
Protein	2	M	2.55	–0.10
Protein	3	M	2.80	0.16
Protein	4	M	2.65	–0.04
Protein	5	M	2.49	0.05
